# Medical News and Miscellany

**Published:** 1898-08

**Authors:** 


### Medical News and Miscellany.

**Dr. D. Leon Sanders** has removed from Elkhart to Edom, Texas.

---

**Dr. E. A. Waldert,** of Tyler, Tex., is spending some weeks in Philadelphia attending the Polyclinics.

---

**Dr. M. A. Taylor,** of this city, was recently married to Mrs. Rathbon, also of Austin. They were married in Wisconsin.

---

**Erratum.**—In referring to the new advertisement of The Lambert Pharmacal Co., Choleraic Diarrhœa, in our last issue the printers made us say "chronic diarrhœa" instead of "choleraic diarrhœa."

---

**The** JOURNAL regrets to state that Dr. C. O. Weller, of Austin, was quite severely hurt July 27th by collision of electric car with his buggy.

Dr. Weller has a son with Roosevelt's Rough Riders, and he was in all the battles around Santiago; a gallant young fellow; known to the New York people as a newspaper man, formerly.

---

**Sharp & Dohme's New Price List.**—We have received the revised price list of Sharpe & Dohme, the well known and popular pharmaceutical manufacturers. Their hypodermic tablets—soluble in two drops of cold water, are standard with up to date physicians. They embrace nearly all desirable combinations—the prescriptions of well known physicians, and represent the potency of most drugs in most concentrated form. A postal card to S. & D. mentioning this notice, will bring a copy of their catalogue free.

---

**For Sale.**—A good country practice,—good collections, and in good farming country. Cost of property will secure practice. A good thing for the right party. Address,

E. C. PARTEE, M. D., Laughlin, Ark.

